# Paper strip-embedded graphene quantum dots: a screening device with a smartphone readout

**DOI:** 10.1038/s41598-017-01134-3

**Published:** 2017-04-20

**Authors:** Ruslan Álvarez-Diduk, Jahir Orozco, Arben Merkoçi

**Affiliations:** 1 Nanobioelectronics and Biosensor Group, Catalan Institute of Nanoscience and Nanotechnology (ICN2), CSIC, The Barcelona Institute of Science and Technology, Campus UAB, Bellaterra, 08193 Barcelona Spain; 2 ICREA, Pg. Lluís Companys 23, 08010 Barcelona, Spain

## Abstract

Simple, inexpensive and rapid sensing systems are very demanded for a myriad of uses. Intrinsic properties of emerging paper-based analytical devices have demonstrated considerable potential to fulfill such demand. This work reports an easy-to-use, low cost, and disposable paper-based sensing device for rapid chemical screening with a smartphone readout. The device comprises luminescent graphene quantum dots (GQDs) sensing probes embedded into a nitrocellulose matrix where the resonance energy transfer phenomenon seems to be the sensing mechanism. The GQDs probes were synthesized from citric acid by a pyrolysis procedure, further physisorbed and confined into small wax-traced spots on the nitrocellulose substrate. The GQDs were excited by an UV LED, this, is powered by a smartphone used as both; energy source and imaging capture. The LED was contained within a 3D-printed dark chamber that isolates the paper platform from external light fluctuations leading to highly reproducible data. The cellulose-based device was proven as a promising screening tool for phenols and polyphenols in environmental and food samples, respectively. It opens up new opportunities for simple and fast screening of organic compounds and offers numerous possibilities for versatile applications. It can be especially useful in remote settings where sophisticated instrumentation is not always available.

## Introduction

Paper is a material of widespread use that was introduced in laboratories since centuries. However, it was not until about one decade ago that it has been rediscovered as a valuable substrate for (bio)sensors development with great potential for field deployment monitoring. Attractive features of cellulosic substrates include biocompatibility, biodegradability, and flexibility, along with easiness of production, functionalization, disposal, and availability worldwide^1–5^. Such properties, together with its intrinsic wicking action, meaning that can drive fluid flow; turn paper an appealing choice to develop inexpensive and disposable sensors and/or integrated sensing platforms. Thus paper-based devices arises as a promising technology for simple, rapid and versatile (bio)sensing^6^.

Nanomaterials combined with paper-based substrates have brought important rewards in the optical sensing field^7–9^. The most widely known assays include dipstick, lateral flow and microfluidic paper-based analytical devices (μPADs)^10^. They typically utilize gold nanoparticles, colored in red due to localized surface plasmon resonance. In these cases, visual detection is achieved through a pronounced color change in gold nanoparticles upon aggregation as a response to assay affinity reactions^11^. Gold nanoparticles enhance sensitivity, stability and cost efficiency to paper-based assays, shifting in this context, their application from limited qualitative yes/no (bio)sensors to sensitive quantitative low-cost devices^12–14^.

Other nanomaterials that are emerging as outstanding probes in the sensing field are photoluminescent semiconductor quantum dots (QDs). These nanomaterials exhibit an excellent brightness, size-tunable photoluminescence (PL), and resistance to photobleaching^8^. Additionally, Inorganic QDs^15, 16^ and QD-containing biocomposites^17^ in combination with Förster resonance energy transfer (FRET) have demonstrated to be suitable for the development of paper-based assays with optical readout. Unlike inorganic QDs, graphene quantum dots (GQDs) have attracted tremendous attention in (bio)sensing, not only because of their higher stability and PL quantum yield but also because of their low cytotoxicity and biocompatibility^18, 19^. GQDs have been proven for the detection of heavy metals^20, 21^, small molecules^22, 23^, and biomacromolecules^24, 25^. It worth to mention that some phenolic compounds as quercetin and nitrophenol have been already quantified by fluorescent quenching of GQDs in aqueous solution^26, 27^. Yet, this will be the first report, that we are aware of, where GQDs and paper are linked together in a paper-based platform.

To date, device readout has typically relied on robust spectroscopic equipment, fluorimeters, and sophisticated fluorescence microscopes, largely proven for sensitive and reproducible laboratory measurements. More portable and inexpensive “scanometric” assay formats, which require only bench top scanners, are being introduced especially as the readout of paper-based devices for point-of-care settings. Recently, smartphone cameras have demonstrated to be a good complement of this technology revolutionizing the way of data recording, data processing, and display readings^28, 29^. Built-in function modules of such portable and ubiquitously available devices are often utilized with multiple purposes going from power source to capturing, controlling or processing data^30, 31^. Indeed, smartphones display real-time and quantitative information, of simple interpretation by the end-user. Integration of mobile phones in (bio)sensing devices is impacting the (bio)sensing field in terms of versatility, portability, significant simplification of designs, reduced sizes and costs of the detecting systems^32^.

This work reports an easy-to-use, low cost and disposable paper-based sensing device for the rapid screening of two groups of phenolic compounds with no required instrumentation other than a simple mobile phone. The screening tool utilizes some luminescent blue GQDs sensing probes embedded into a nitrocellulose matrix. The probes were synthesized from citric acid by a pyrolysis procedure with no modifications. Further, they were physisorbed and confined within small wax-traced spots on the nitrocellulose substrate. A UV LED that excites the GQDs was placed within a homemade 3D-printed dark chamber. A mobile phone was used as both, energy source and digital color imaging capture. The dark chamber isolates the paper platform from external light fluctuations leading to highly reproducible data. The nitrocellulose-based device seems to be a promising tool for (i) screening antioxidant capacity related with flavonoid content in wine samples and (ii) screening 4-nitrophenol and paraoxon pollutants in seawater samples. The generated quantitative data open new opportunities for a rapid, simple and in field screening of such organic compounds. Nevertheless, of more interest should be placed on the numerous possibilities offered by the disposable paper-based analytical device. It has versatile applications, of special utility in low-income or remote settings where more expensive and sophisticated equipment is not always possible or not available.

## Results and Discussion

The paper-based screening device proposed in this study, consists of PL-GQDs sensing probes contained within the 3D structure of a nitrocellulose matrix. Paper was selected as substrate due to its superior ability to wick aqueous solutions by capillary action, among other recognized qualities that make pumps or high power source requirements not necessary^33^. The sensing mechanism observed here is based on fluorescence resonance energy transfer where the GQDs fluorescence quenching occurs due to its interaction with some phenolic compounds.

**Figure 1:**
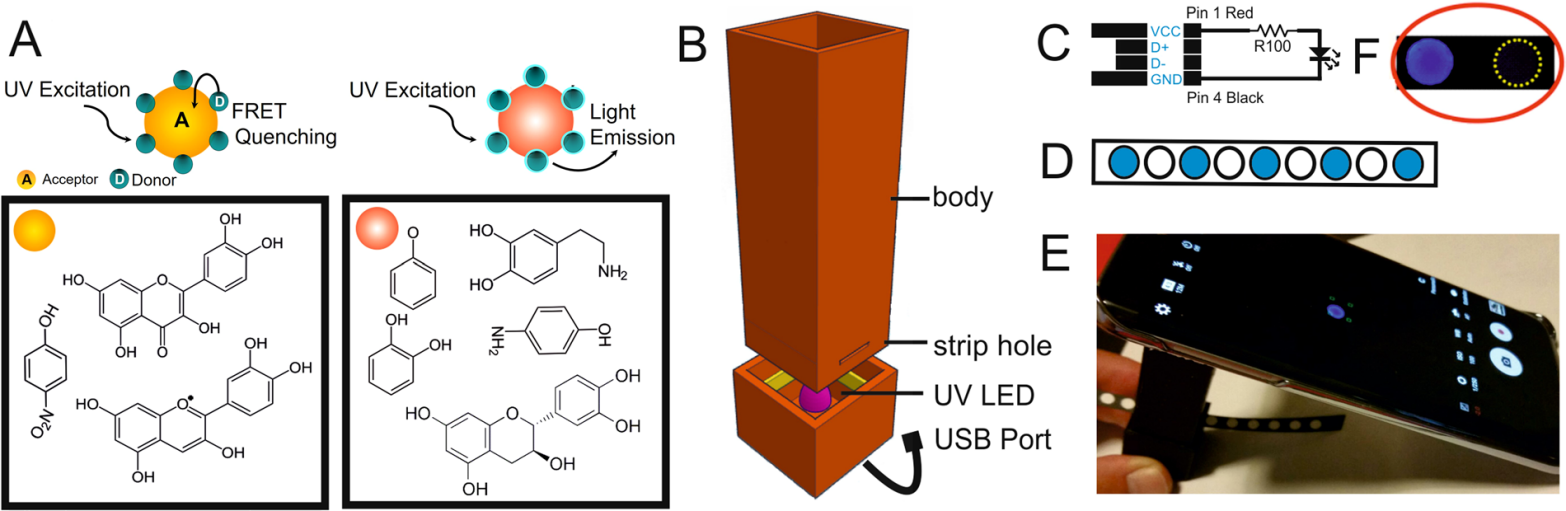
Schematic representation of the fluorescence quenching of Quantum Dots in the presence of an analyte by the FRET phenomenon. (**A**) Examples of phenolic compounds susceptible of producing GQDs quenching and those unable to produce it are denoted with a yellow and red circle, respectively. (**B**) Schematic representation of a 3D-printed device with its different parts. (**C**) The electric circuit of the 365 nm UV LED connected to the male USB port. (**D**) Scheme of a nitrocellulose paper strip with wax-printed circular areas. (**E**) Image of the sensing platform where the fluorescent spot is observed in the middle of a mobile phone screen. (**F**) Picture of the sensing area of a yes/no (ON/OFF) typical result.

Figure 1A (left) illustrates the FRET phenomena. Phenolic compounds able to produce GQDs quenching are colored in yellow. Excited-state photons from the GQDs are transferred to the analyte displaying a highly efficient quenching of the fluorescence. The opposite is showed in Fig. 1A (right) where phenolic compounds, although structurally similar, are unable to quench (red color) due to the interruption in the conjugation of the aromatic ring. As a consequence, the absorption spectrum is different from compounds in the left side and the quenching is not observed. The GQDs were synthesized by the simple pyrolysis of citric acid^34^ and pipetted on top of the paper strip, defining in this way, the desired sensing spots (Fig. 1D). Samples along with reagents were placed at the delineated area where the sensing later occurs. The quenching of GQDs is the evidence of a positive sensing event that is captured by a smartphone camera and quantified by a software treatment. For this purpose, the strip is first placed within a homemade 3D-printed dark chamber (Fig. 1B); see details in the experimental section. The dark chamber contains a strip hole where the paper strip goes through. Each spot is processed, one at the time, to reach the UV LED area. The LED excites the confined GQDs at 365 nm. This LED is powered by a smartphone through an USB port utilizing the simple circuit schematized in Fig. 1C. The smartphone captures the light that the GQDs emit at 460 nm as shown in Fig. 1E. The amount of captured fluorescent light is further related to the concentration of the analyte, which can be quantified after processing by the imageJ program. Figure 1F depicts a sensing area of a yes/no (positive/negative) result as a typical example. More details of the dark chamber and assembled sensing setup are depicted in supporting information Figure S1.

**Figure 2:**
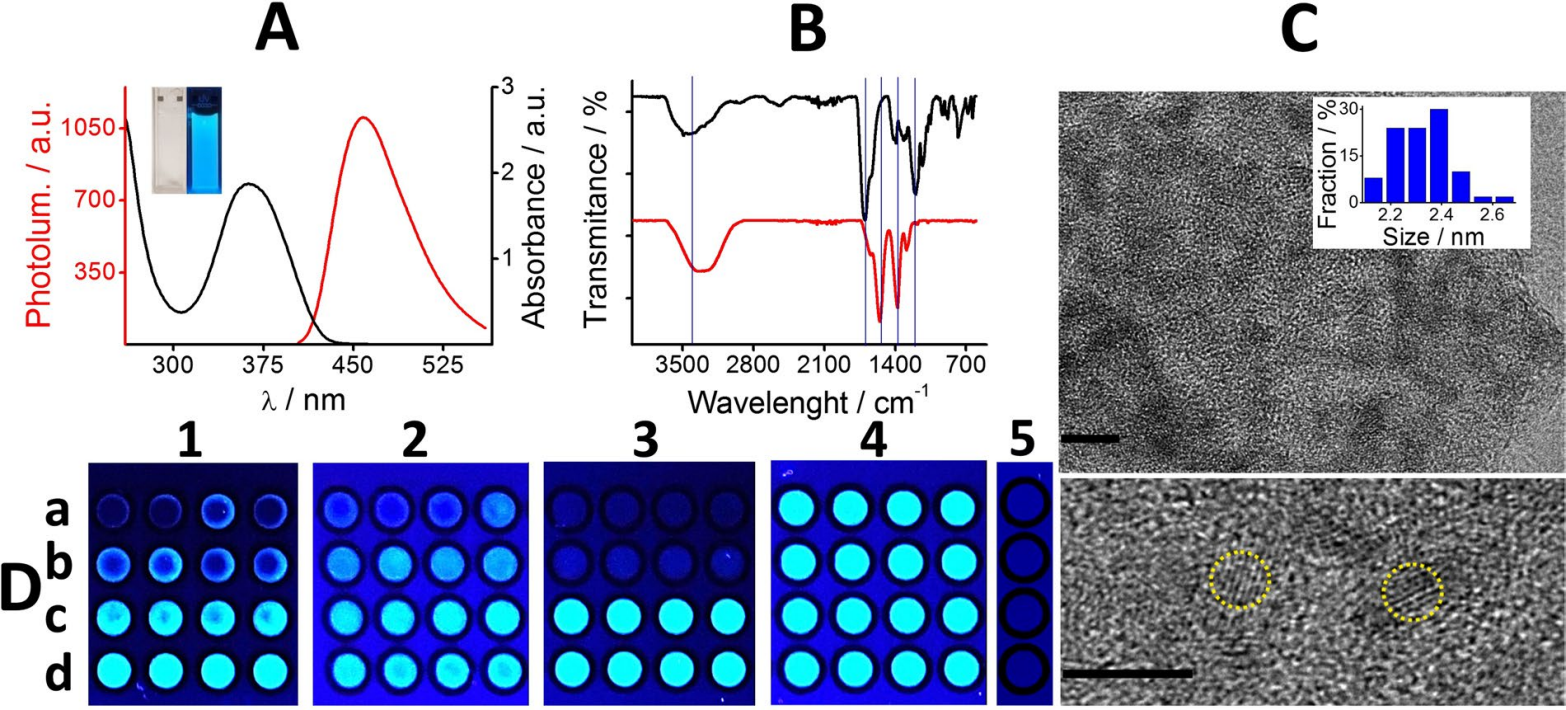
Characterization of the GQDs and the GQD-modified strip. (**A**) Absorbance (black line) and photoluminescent (red line) spectra. Iinset, a picture of the GQDs under UV (right) and visible (left) light, respectively. (**B**) FT-IR spectrum of citric acid (black line) and GQDs (red line), respectively. (**C**) TEM image (upper and bottom scale bars are 5 nm, respectively). Inset is the plot of particles size distribution. GQDs lattice planes are denoted by yellow circles. (**D**) Optimization of the GQDs-modified paper-based sensing platform in terms of fluorescence intensity and homogeneity of the sensing area. (1) Concentration of GQDs solution (a) 6.25, (b) 12.5, (c) 25 and (d) 50 mg/ml. (2) Volume of GQDs solution, (a) 1, (b) 2, (c) 3, and (d) 4 µL. (3) GQDs pH value, (a) 4, (b) 6, (c) 8, (d) 13. (4) Number of additions of a volume of 4 µL, (a) 1, (b) 2, (c) 3, and (d) 4 times. All of them placed into the wax-patterned areas from the nitrocellulose support. The concentration of GQDs solution is always 50 mg/ml except in (a). (5) 0 mg/ml GQDs (from a to d) used as control to discart other possible interferences. Experiments were performed in quadruplicate and represented in each row (a–d).

Once synthesized, the GQDs were further characterized by spectrophotometry, fluorimetry, Fourier transform infrared (FT-IR) spectroscopy and high-resolution transmission electron microscopy (HRTEM) imaging. Figure 2A shows both, the UV-visible absorbance spectrum of GQDs (black line) characterized by a wide pick between 300 and 430 nm and the photoluminescence spectra with a strong fluorescent emission peak at 460 nm (red line) when they are excited at 365 nm. These data are in agreement to those reported in the literature for GQDs^34^. The inset depicts a picture of the GQDs under UV (right) and visible (left) light, respectively. Figure 2B overlaps the FT-IR spectra of the as-synthetized GQDs (red) and its citric acid precursor (black) showing their different chemical structure. For example, the broadband centered around 3300 cm^−1^ from the GQDs respect to the one around 3430 cm^−1^ from its precursor are associated with absorption of carboxyl and hydroxyl groups. The bands at 1730 and 1200 cm^−1^ are attributed to the C=O and C–O stretching vibrations of carboxylic acid groups, respectively. The doublet bands at 1550 and 1370 cm^−1^ observed only in the GQDs spectrum correspond to the COO^−^ symmetric and asymmetric vibrations. These results are in agreement with previously reported studies for GQDs^34^. HRTEM image depicted in Fig. 2C shows the morphology of fresh synthesized GQDs. The image depicts well-dispersed GQDs with a circular shape and an average size of 2.33 ± 0.12 nm (n = 50). The presence of lattice planes, noticeable in the HRTEM image, is an indication of a high crystallinity of the as-prepared GQDs. The measured interplanar distance is around 0.26 nm which is in agreement with data reported by Ge and collaborators^35^.

**Figure 3:**
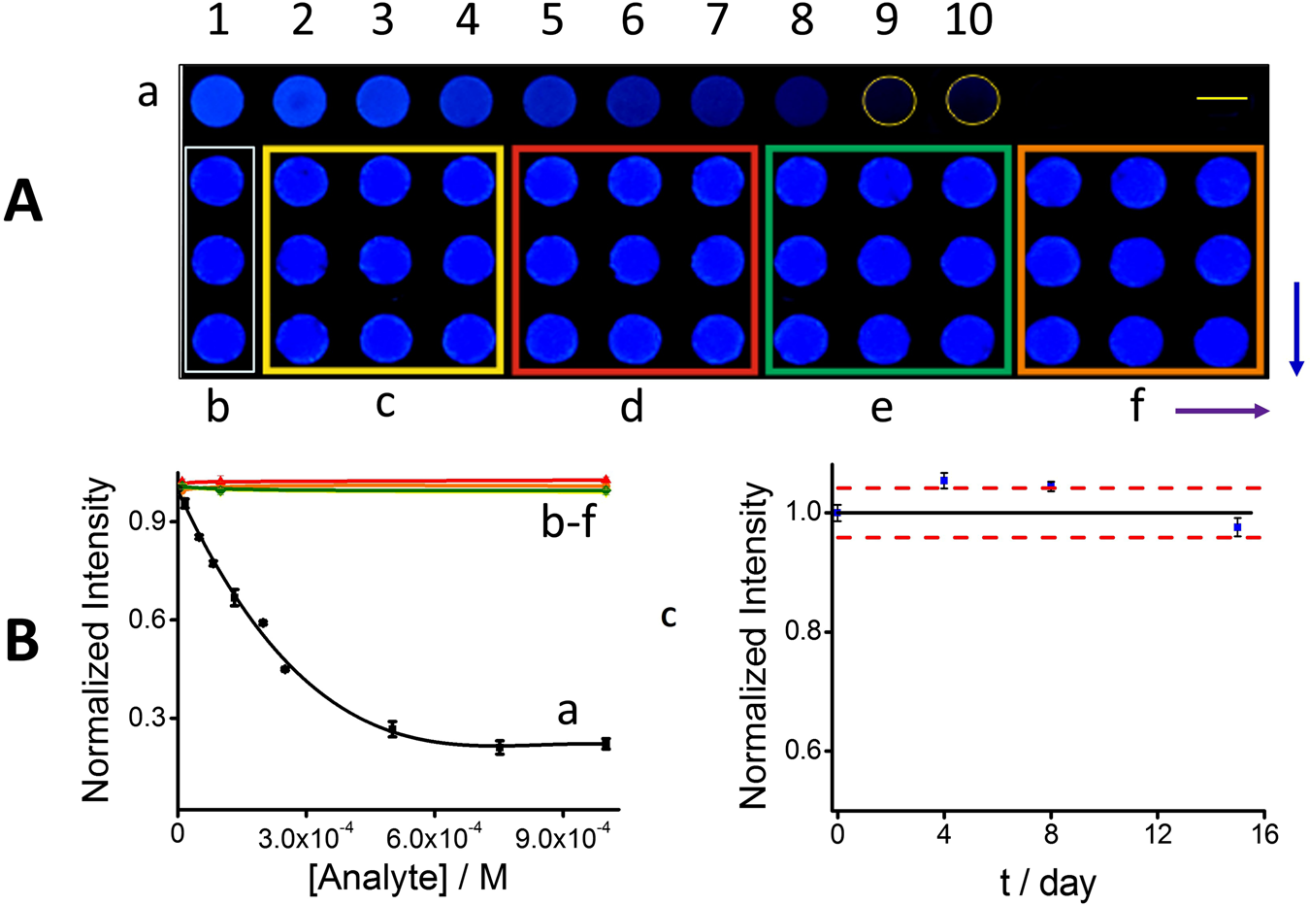
GQDs-modified paper sensing platform. (**A**) Picture of GQDs-modified nitrocellulose paper under UV light. (a) Fluorescence quenching at different concentrations of quercetin: 0 (0.01 M PBS buffer), 1.66 × 10^−5^, 4.98 × 10^−5^, 8.30 × 10^−5^, 1.33 × 10^−4^, 1.99 × 10^−4^, 2.51 × 10^−4^, 5.01 × 10^−4^, 7.52 × 10^−4^, 1.25 × 10^−3^, 1.50 × 10^−3^ M (from 1 to 10), respectively. Selectivity testing: (b) 0.01 M PBS buffer and increasing concentrations (per each square as indicated by the purple arrow) of (c) phenol, (d), catechol (e) hydroquinone and (f) 4-aminophenol, 1.00 × 10^−5^, 1.00 × 10^−4^ and 1.00 × 10^−3^ M, respectively. The blue arrow indicates the same concentration by triplicate. Yellow dotted lines define the sensing area in cases where was difficult to determine, for instance, at a high extent of quenching. Scale bar is 4 cm. (**B**) Corresponding normalized intensity-analyte concentration profile with fluorescent intensity obtained from (**A**). (**C**) Stability testing of the paper-based sensing platform upon time: control chart based on the average intensity for the first day (continuous black line) and following the ± 3σ criterion (dashed red lines).

**Figure 4:**
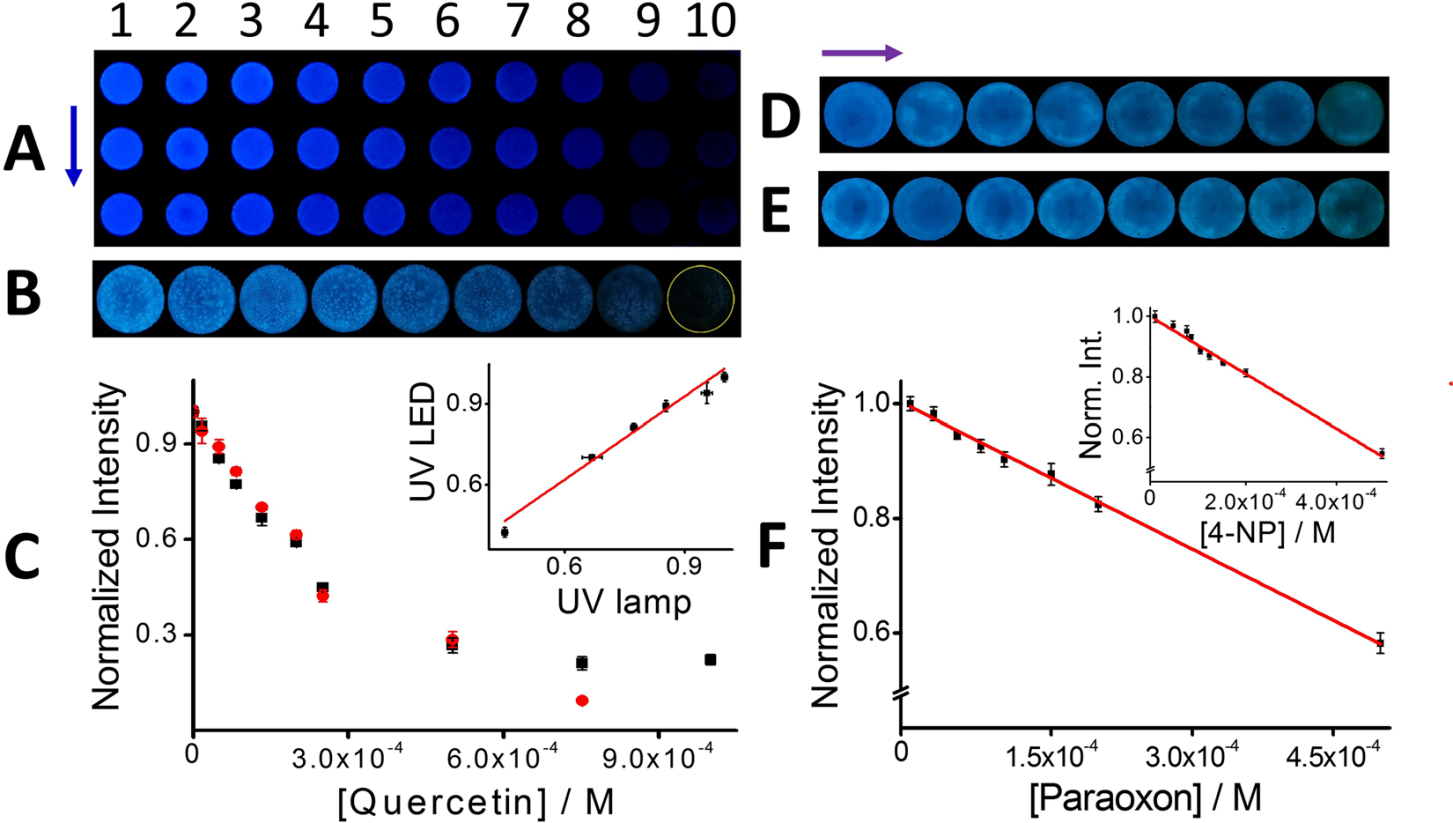
Performance of the LED-based device compared to the UV light readouts. Fluorescence quenching of GQDs with increasing concentrations of quercetin observed under the UV light (**A**); and with the LED device (**B**), respectively. Concentrations are 0 (0.01 M PBS Buffer), 1.66 × 10^−5^, 4.98 × 10^−5^, 8.30 × 10^−5^, 1.33 × 10^−4^, 1.99 × 10^−4^, 2.51 × 10^−4^, 5.01 × 10^−4^, 7.52 × 10^−4^, 1.25 × 10^−3^, 1.50 × 10^−3^ M, from 1 to 10, respectively. Three replicates of the same concentrations are denoted by the blue arrow. Graphics of estimated fluorescence of the spots with increasing concentration of quercetin observed under the UV lamp (black dots); and with the LED device (red dots) (**C**). Inset is the correlation of the two methods. Decreasing concentrations of 4-nitrophenol (4-NP) (**D**) and paraoxon (**E**) ranging from 0 to 5 × 10^−4^ M are denoted by the purple arrow. The corresponding Stern–Volmer relationship for paraoxon (**F**) and 4-NP (inset).

**Figure 5:**
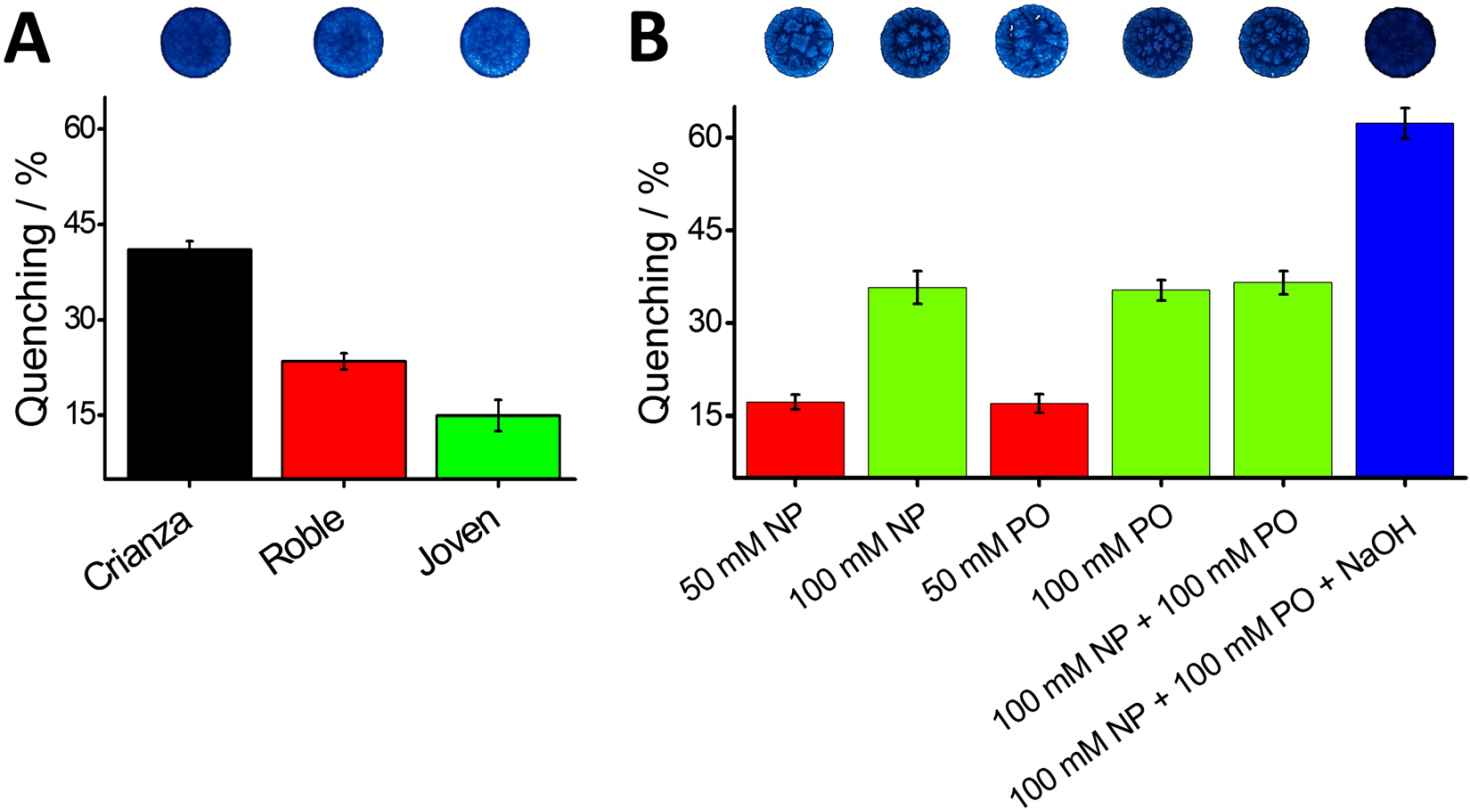
Interrogation of the GQDs-modified paper-based sensing platform in food and environmental samples. Fluorescence quenching of the paper-based sensor in response to (**A**) wine samples of different vintage and (**B**) seawater samples spiked either with 50 or 100 mM 4-Nitrophenol (NP) and Paraoxon (PO), 100 mM NP and 100 mM PO and 100 mM NP, 100 mM hydrolyzed PO (1 M NaOH). Tempranillo wine samples were diluted (1/10) in PBS at pH 7.4 and seawater samples were tested as received. All quenching values presented were normalized regarding the blank signal (maximum fluorescence GQDs emission without analyte, see details in experimental section).

After synthesis and characterization, the GQDs were placed on top of the nitrocellulose support, selected after preliminary tests from different cellulosic substrates, including Whatman chromatographic- and bacterial cellulose-paper. Nitrocellulose features such as, high surface-to-volume ratio, resistance, easiness of handling and higher capabilities of GQDs confinement were responsible for its selection. The substrate turns out to be ideal for the assay where the sensing probes were bound to paper interstices and reagents (if needed) together with samples placed on its surface. So far, wax-printing is the most convenient method for paper patterning^36, 37^ therefore, in order to confine the sensing probes, wax-printed hydrophobic circular patterns were previously defined on the nitrocellulose matrix. The GQDs were physically adsorbed into it while the barrier contained the fluorescent probes in the as-defined area. The first set of experiments was conducted to optimize the GQDs-modified paper-sensing platform in terms of fluorescence intensity and homogeneity of the sensing area. The first test consisted of confining GQDs from solutions of different concentrations ranging from 6.25 to 50 mg/ml. Figure 2D-1a–d shows that for lower concentrations of GQDs the “coffee ring effect” is obvious. This pattern is expected from the puddle of GQDs particles-laden liquid after its differential evaporation. Moreover, the higher the GQDs concentration, the higher the luminescence (a–d). These results demonstrate the direct dependence of the level of luminescence on GQDs concentration. Towards an optimal sensing platform, the volume of GQDs solution was also varied from 1 to 4 µL (Fig. 2D-2a–d). Smaller volume led to significantly lower fluorescence intensity (a and b) while higher volume results in a more homogeneous dispersion of GQDs in the sensing area. GQDs pH value showed to be the most crucial parameter to be controlled. While at pH 4 and 6 (Fig. 2D-3a and b) protonated GQDs showed to dramatically quench the fluorescence, the deprotonated GQDs at pH 8 and 13 (c and d) were absolutely fluorescent. However, multiple additions of the same volume of GQDs from 1 to 4 times (Fig. 2Da–d) did not lead to any improvement of the sensing platform in terms of better dispersion or higher luminescence. 0 mg/ml GQDs (Fig. 2D-5, from a to d) were used as control to discart other possible interferences. Based on the aforementioned results, 4 µl of 50 mg/ml GQDs solution was added once to the wax-delineated sensing area letting it dry before testing it as a sensor in further experiments.

The sensing potential of the nitrocellulose-embedded GQDs strip was tested first with quercetin as a model phenolic compound of double bonds conjugated within multiple aromatic rings. Quercetin is a flavonoid, naturally present in some foods, grains, leaves and beverages (including wines) with known antioxidant properties^38, 39^. Its molecular structure contains five –OH groups conjugated with the double bonds of its three aromatic rings. Figure 3A shows a picture of the GQDs-modified paper platform under UV light. The fluorescence quenching was higher as concentrations of quercetin buffered solutions were increasing in a range of two orders of magnitude, spots from 2 to 10 respectively. Quenching of the fluorescence was negligible in spot 1 with only PBS buffer. Based on our experiments, at the pH value of 9, the deprotonated quercetin and GQDs have shown an efficient quenching of the GQDs.

Although several mechanisms have been proposed to explain the fluorescence quenching of QDs^26^, it has not been completely elucidated. Indeed, there are often conflicting reports about the GQDs intrinsic behavior. To propose the GQDs quenching mechanism, we systematically evaluated a series of compounds with structural and/or functional quercetin analogy (see Table S1). Polyphenolic compounds with full conjugation throughout the entire molecule caused different levels of GQDs quenching, when tested at the same concentration (morin > myricetin > quercetin > kamepherol) (see quenching extent in Figure S2). Compounds with the same amount of hydroxyl groups as quercetin but with the conjugation between the two rings interrupted (e.g. catechin and hesperetin) did not produce any quenching on the GQDs. In the same way, fully conjugated compounds without hydroxyl groups in their structure did not produce a fluorescent decrease either (carotene and xanthone). It has been recently reported that energy emission spectrum from the donor fluorophore and energy absorption spectrum of the acceptor must overlap for a FRET quenching mechanism^40^. When we analyzed the spectra of the analogous flavonoids at a fixed pH of 7.5, we indeed observed overlapping of their absorption bands and the GQDs emission band (Figure S3). Although deeper studies would be needed to elucidate the quenching mechanism, which is out of the scope of this paper, our results show that the FRET is the more likely quenching mechanism. In addition, the extent of quenching increases as the area of overlapping increases (Figure S2).

Other phenolic compounds did not show any quenching of the QDs neither in aqueous solutions nor in the paper-based sensing platform (Figure S2, red bars), as expected. For instance, a phenol with one –OH group and one aromatic ring only Fig. 3A (c); catechol and hydroquinone, containing 2 –OH groups but only 1 aromatic ring, (d and e) respectively and 4-aminophenol (f), containing 1 –OH group and one –NH_2_ group (of nucleophilic character). Unlike quercetin, none of the last mentioned related compounds showed quenching capabilities. Spots with only PBS buffer were also tested as control (a) with not quenching. These results, support that FRET is the more likely quenching mechanism. Figure 3B shows the resultant normalized intensity-quercetin concentration profile (a) regarding to phenol, catechol, hydroquinone, and 4-aminophenol, respectively (b–f); fluorescence intensity was estimated after image processing (see details in the experimental section). While the maximum quenching of GQDs riches 80 ± 2% (at 7.5 × 10^−4^ M quercetin), the same concentration of the related compounds tested produces a negligible quenching. Stability of the paper-based sensing platform was evaluated upon time based on the ± 3σ criterion (Fig. 3C). For this purpose, we plotted a control chart with the average intensity of the sensing area for the first day of the study as central value (continuous black line), setting the lower and higher control limits at three times the standard deviation of this value (dashed red lines). Figure 3C shows the estimated fluorescence was still within the control chart even after 15 days of storage at 4 °C, demonstrating the stability of the paper-based platform.

At this point, we have demonstrated that our paper-based strip has sensing properties able to quantify quercetin in highly reproducible laboratory measurements. Indeed, the GQDs fluorescence plot in a solid platform (paper) correlate well with experiments performed in aqueous solutions using a fluorimeter (see supporting information, Figure S4). They fit the equation y = 0,006e^5,2874*x*^ with a correlation coefficient, r^2^ = 0.9723. Although, the exponential relation between them reflects the lower fluorescence of the GQDs within the paper sensor, such apparent disadvantage expects to be overcome by its great potential for fast and inexpensive field deployment monitoring, as mentioned before. The analytical information was extracted from pictures taken with a smartphone camera after illuminating the sensing areas with a simple UV light lamp. The widely spread smartphones, serving as rapid and ready data recording and data processing device well-complemented this approach. Consequently, in order to demonstrate the potential of the platform to get reproducible data in outdoor settings, foreseeing that there may be external light fluctuations, we developed a 3D-printed homemade dark chamber to isolate the paper platform from external light (see details in the experimental section). To validate the homemade chamber, we compared results of the fluorescence quenching of the paper platform with increasing concentrations of quercetin with the readout based on the UV light lamp (Fig. 4A) and with those from the UV LED system (Fig. 4B). The resultant normalized intensity-analyte concentration profile showed not only a high reproducibility of results but also no statistically significant differences between the two methods up to 5 × 10^−4^ M (p < 0.001). From data, the linear range of concentration was from 1.66 × 10^−5^ to 1.33 × 10^−4^ M for the UV light lamp-; and from 1.66 × 10^−5^ to 2.50 × 10^−4^ M (slightly improved) for the UV LED-methods (Fig. 4C, black squares and red circles), respectively. Furthermore, the Stern–Volmer plots correlate well with the equation Fluorescence intensity_*UV lamp*_ = −2522,3 [quercetin] + 0,9925, with a regression coefficient, r^2^ = 0.9934; and Fluorescence intensity_*UV LED*_ = −2265,3 [quercetin] + 0,9966, with a regression coefficient, r^2^ = 0.9977. LOD was estimated to be 6.67 × 10^−5^ and 2.35 × 10^−5^ M for the readout based on the UV light lamp and the UV LED, respectively. The set of measurements with the two different methods are correlated by the equation y = 1,02 × −0,0056, with r² = 0,9805. Those results indicate that the UV LED integrated within the 3D-printed dark chamber can potentially balance external light fluctuations coming from outdoors settings, with similar expected results respect to those in laboratory settings. It is important to note that it is required to have a mobile phone camera with manual mode configuration, otherwise the auto mode will adjust the parameters to compensate the light differences with no possibilities of detecting the emission decrease. (See the experimental section for more details).

To explore better the sensing capabilities of the paper-based platform we tested the ability of the nitrocellulose-embedded GQDs with another group of phenolic compounds of environmental interest. For example, 4-nitrophenol (4NP) has shown to quench GQDs in aqueous solutions^27^. In this context, we examined whether nitrocellulose-embedded GQDs strip irradiated by a simple LED and coupled only to a smartphone camera is able to provide a sensitive response to this type of pollutants. Unlike polyphenols, 4NP has oxidant properties and exhibits different levels of quenching depending on the pH. At pH = 6, close to the pKa^41^, 4NP species coexist protonated and deprotonated producing a lower quenching compared to the totally deprotonated molecule at pH = 9 (see Figure S5). Absorption spectra of 4NP at the different pH values show an absorption band at about 410 nm that increases as the pH increases^41^, thereby increasing the overlapping with the emission band of the GQDs. The fluorescence intensity of the GQDs at different pH values (6, 7.5 and 9) is shown in Figure S6. These results are in agreement with the FRET-based GQDs quenching mechanism. At the proper pH, deprotonated 4-nitrophenolate molecules work as acceptors of energy while GQDs are the donors. At basic pH, given by a 1 M NaOH solution, hydrolyzes of the paraoxon pesticide produces 4-nitrophenol as bi-product. The as-developed paper-based platform demonstrated capabilities as sensor not only of 4-nitrophenol but also hydrolyzed paraoxon. Figure 4 shows how fluorescence decreased as the concentration of 4-nitrophenol (D) and paraoxon (E) increased from 2.5 × 10^−5^ to 5 × 10^−4^ M (in the direction of the purple arrow). Figure 4F shows the corresponding paraoxon Stern–Volmer plot, which correlates well with the equation Fluorescence intensity_*UV LED*_ = −827.84 [paraoxon] + 0,9939, with a regression coefficient, r^2^ = 0.9972. In the inset, the corresponding linear correlation of 4-nitrophenol that fits well with Fluorescence intensity_*UV LED*_ = −910.10 [4-nitrophenol] + 0,9959, with a regression coefficient, r^2^ = 0.9912. LOD were estimated to be 4,36 × 10^−5^ and 3,97 × 10^−5^ M for paraoxon and 4-nitrophenol, respectively (3σ criteria). These results demonstrate the potential of the newly developed sensing paper platform for environmental monitoring.

The final experiments were devoted to demonstrate the practical utility of the sensing platform in food and environmental samples (Fig. 5). For this purpose, Tempranillo wine samples of different vintage were investigated with the GQDs-modified paper sensor (panel A). The older the wine, the higher the fluorescence quenching extent. For instance, the ‘crianza’ wine led to a 41.1 ± 1.3% quenching while ‘roble’ and ‘joven’ wines only 23.4 ± 1.3 and 15.0 ± 2.4%, respectively. Although inconclusive, these results allow to relate the wine antioxidant phenolic content with the age based on the comparison of vintage wines to younger wines, which is in agreement with previous studies^42^. Results suggest that the sensor strip holds potential for a rapid characterization of wines based on their antioxidant capacity, which could be exploited in other beverages and foods. Sensing capabilities of the paper-based strip were also tested in environmental samples. A seawater sample (panel B) was spiked with 50 and 100 mM NP producing a quenching of the GQDs of 17.1 ± 1.2 and 35.7 ± 2.6%, respectively. 50 and 100 mM hydrolyzed paraoxon (PO) led to similar quenching extents (16.9 ± 1.5 and 35.3 ± 1.7, respectively) as expected from the 4-NP, stoichiometrically produced by the hydrolysis of PO. The mixture of 100 mM NP and 100 mM unhydrolyzed PO produced only 36.5 ± 1.9% of quenching whereas almost twice this value was reached (62.3 ± 1.9%) when the hydrolyzed mixture was simultaneously tested. The corresponding sensing areas are in the upper part of Fig. 5. These results demonstrate that the paper-based device can be used for in field monitoring of 4-NP and PO separately and when they coexist in an environmental sample.

In summary, we have developed an easy-to-use, low-cost and disposable paper-based device for the rapid screening of two groups of phenolic compounds in food and environmental samples. The paper strip is embedded with fluorescent GQDs, introduced in a 3D-printed homemade dark chamber and irradiated with UV light from a LED. The fluorescence of the GQDs was dramatically quenched by interaction with the analyte. The resultant quenching was registered by a mobile phone camera and fluorescent counts extracted from. High yield of the GQDs along with outstanding features of paper platforms and the smartphone readout were exploited in the development of the presented device, towards a new generation of rapid, inexpensive and simple paper-based analytical tools with versatile applications. The response of the device correlated well with the flavonoid content of wine samples of different vintage; but could be exploited in other beverages and foods. Quenching of fluorescence also correlated well with changes in the concentration of 4-nitrophenol and paraoxon pollutants separately or coexisting in seawater samples. Overall results are promising for monitoring in settings where sophisticated instrumentation is not always available.

## Materials and Methods

### Reagents

Citric acid, saline phosphate buffer (PBS), phenol, hydroquinone, quercetin, catechol, kaempferol, morin, myricetin, mangiferin, curcumin, 1,4-benzoquinone, vanillic acid, gallic acid, ascorbic acid, phenol, catechin, hesperetin, cyanidin, β-carotene, xanthone, hydroquinone, ibuprofen, amoxicillin, dopamine, caffeic acid and 4-nitrophenol were purchased from Sigma. Sodium hydroxide and ethanol were purchased from Panreac and 4-aminophenol from Fluka. Stocks solutions of each analyte were prepared in ethanol and stored at room temperature when not in use, while dilutions were prepared in MilliQ deionized water. All reagents were at least analytical grade. The nitrocellulose paper was purchased from Millipore (HFC1800425). Whatman grade 1 chromatographic- and bacterial cellulose-paper were purchased from GE Healthcare, Life Sciences, and Nano Novin Polymer Co, respectively.

### GQDs Synthesis

Graphene oxide quantum dots were synthesized according to the procedure reported by Yongqiang Dong *et al*.^34^. Briefly, 1 g of citric acid was pyrolyzed at 200 °C until the color changed from transparent to orange in approximately 30 min. Then, the orange liquid was slowly transferred to a 50 ml 10 mg/ml NaOH solution under vigorous stirring. The stock suspension of GQDs was adjusted with 1 M HCl to get 50 mg/ml GQDs solution with a pH value bellow 9 and kept in the fridge at 4 °C.

### Paper strip preparation and set up

Wax circumferences of 4 or 5 mm of diameter and 0.5 mm of width were printed on top of a nitrocellulose paper strip (9 mm × 20 mm) with a Xerox ColorQube 8570 solid ink printer. Afterward, the strip was placed on a hot plate at 84 °C for 2 minutes melting the wax and creating an internal 3D-wax structure confining spot. Then 4–8 µL of the as-prepared GQDs were added to the spots; and let it dry at room temperature for approximately 45 min. The paper strip was placed either under the UV lamp or within the homemade 3D-printed dark chamber. Either a 20 W UV lamp (HQ Power vdl20uv) or a 5 mm LED of 20 mA at 365 nm wavelength was used as an UV source (the LED introduced into the 3D-printed dark chamber). All the pictures were taken with a Samsung Galaxy S7, in manual mode, autofocus; at ISO 100 and shooter speed 1/180 s. Images were analyzed either with Image J, or directly from the camera using “IJ Mobile” App.

### Sensing experiments

4–8 µL of the analyte at different concentrations were added to the as-prepared fluorescent sensing area in the paper strip. After drying at room temperature, pictures of different spots were taken with the smartphone adapted to the dark chamber. Photoluminescence was estimated from the pictures. Wine samples were diluted 1/10 with PBS pH 7.4, which final ethanolic content was 15%. Seawater samples were spiked with the desired concentration of 4-nitrophenol or paraoxon and the resultant solution containing 5% ethanol without dilution was tested. The seawater was collected from the beach of “La Barceloneta” (41°22′34.4″N 2°11′29.8″E) and tested without any further dilution.

## Electronic supplementary material

Supporting information
