# Supplementary material for: Paper strip-embedded graphene quantum dots: a screening device with a smartphone readout
Source: Sci Rep. 2017 Apr 20;7:976. doi: 10.1038/s41598-017-01134-3 (PMC5430532; doi:10.1038/s41598-017-01134-3)
Supplement: Supplementary file 1 — Supporting information [file 41598_2017_1134_MOESM1_ESM.pdf]

Supporting Information

Paper strip-embedded graphene quantum dots: a screening device with a smartphone readout.

Ruslan Álvarez-Diduk[a], Jahir Orozco[a], Arben Merkoçi[a,b] *.

[a] *Nanobioelectronics and Biosensor Group, Catalan Institute of Nanoscience and Nanotechnology (ICN2), CSIC. The Barcelona Institute of Science and Technology, Campus UAB, Bellaterra, 08193, Barcelona, Spain.*

[b] *ICREA, Pg. Lluís Companys 23, 08010 Barcelona, Spain.*

Table S1. Aromatic compounds and their molecular structure used to proof the fluorescence quenching of GQDs in aqueous solutions.

**Compounds that produce fluorescence quenching**			
**Compound**	Structure	Compound	Structure
**Morin**	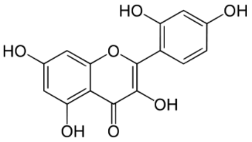	Cyanidin	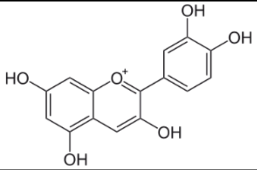
**Myricetin**	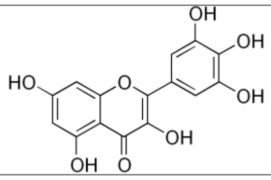	Mangiferin	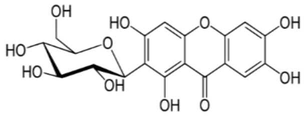
**Kaempherol**	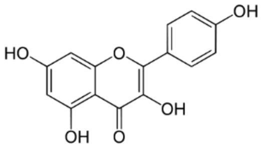	Curcumin	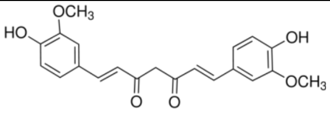
**Quercetin**	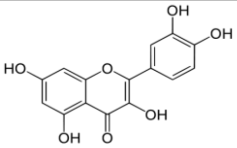	4-Nitrophenol	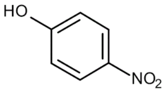
**Compounds that don´t produce fluorescence quenching**			
**Compound**	Structure	Compound	Structure
**Catechol**	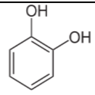	Hesperetin	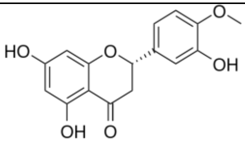
**Hydroquinone**	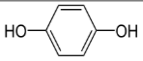	Catechin	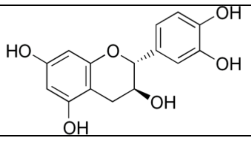

**Phenol**	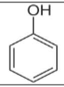	β-Carotene	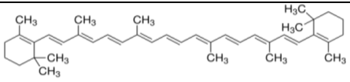
**Ascorbic acid**	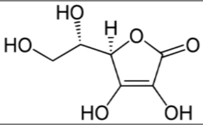	Genistein	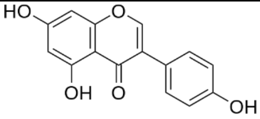
**Dopamine**	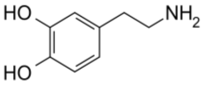	Xanthone	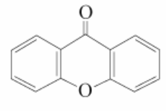
**Ibuprofen**	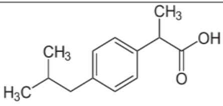	Gallic acid	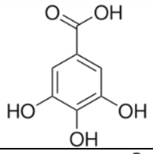
**Amoxicillin**	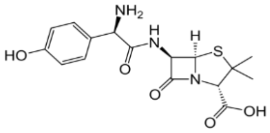	Caffeic acid	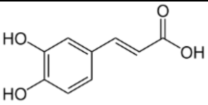
**Rosmarinic acid**	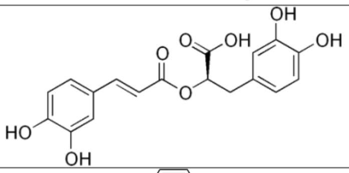	Vanillic acid	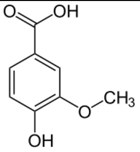
**1-4 Benzoquinone**	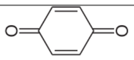	4-Aminophenol	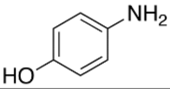

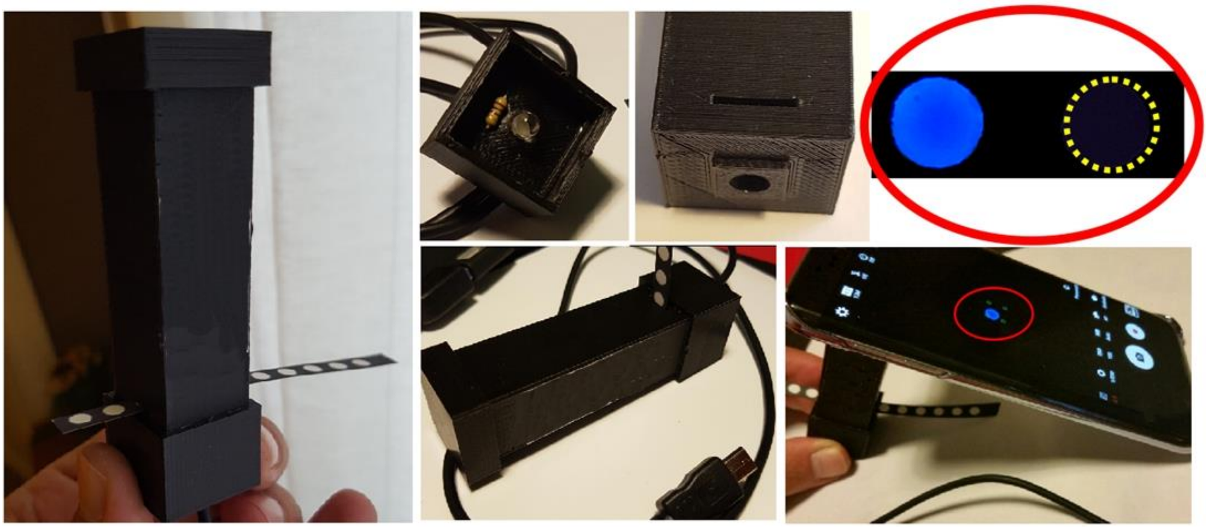

Figure S1. Details of the black chamber and assembled sensing setup.

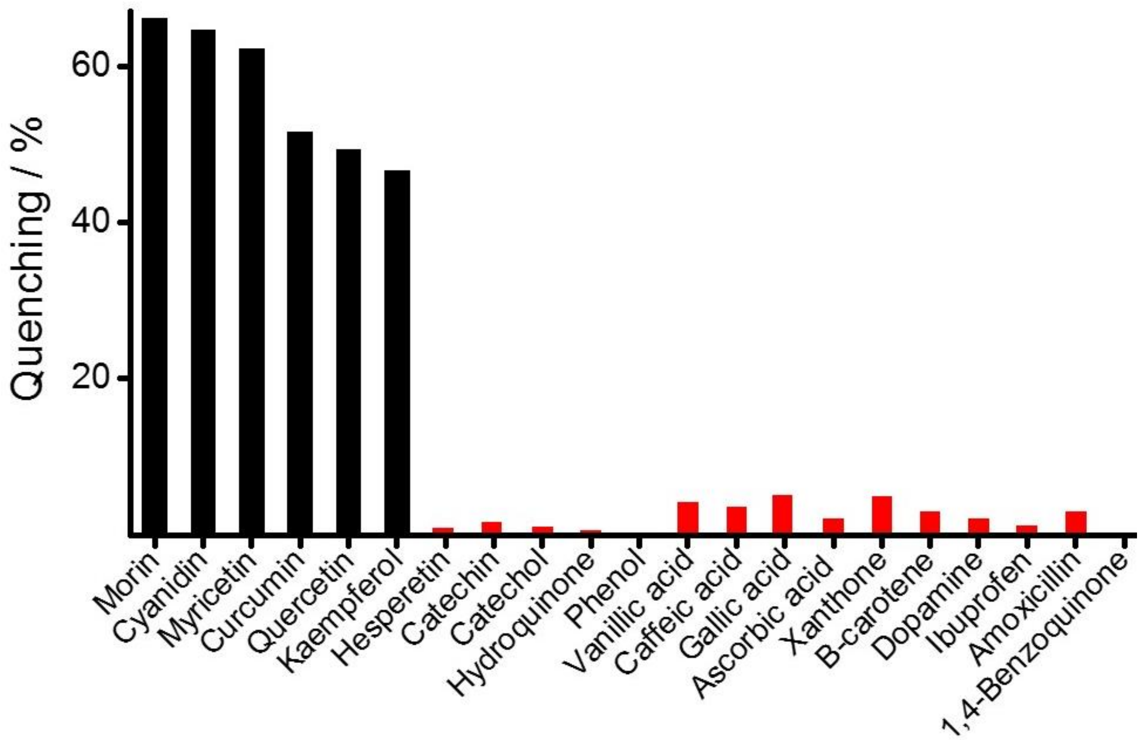

Figure S2. Extent of quenching of the paper platform-embedded GQDs as a response to phenolic and polyphenolic compounds. Concentration of the different analytes was always 1 x 10^{-5} M.

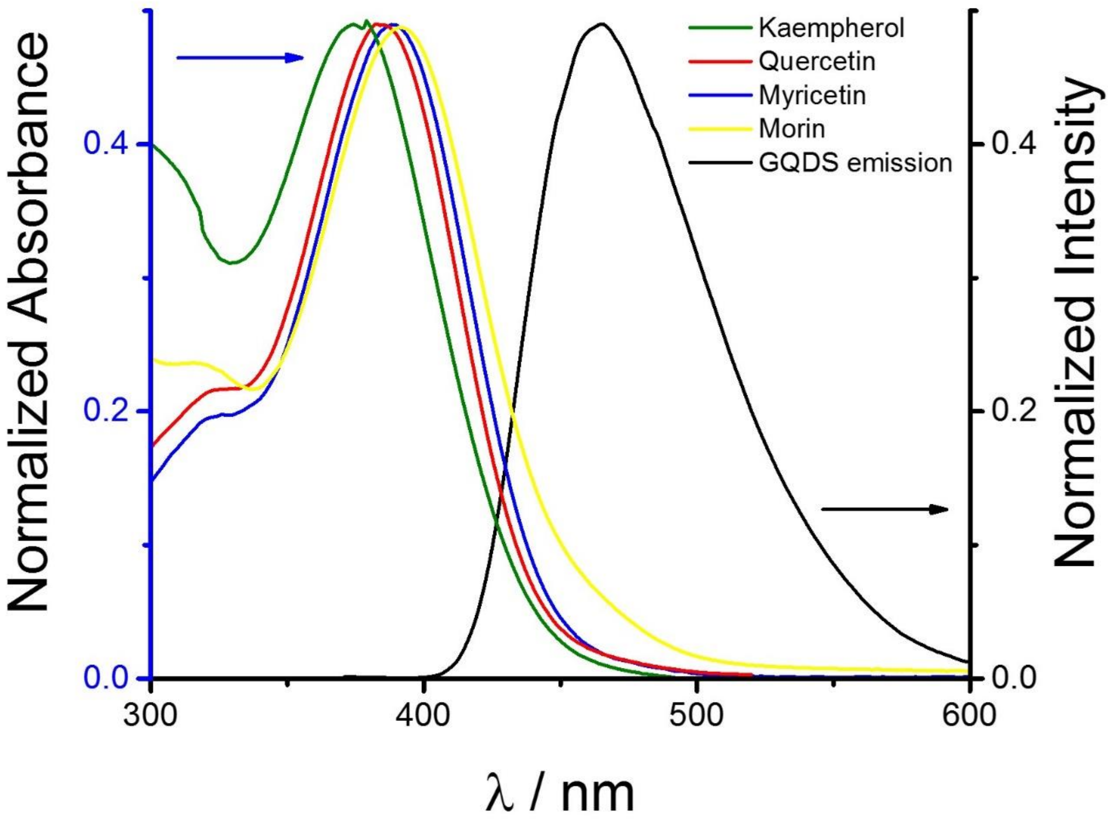

Figure S3. Overlapping of the absorbance spectrum bands of morin, myricetin, quercetin and kaempherol with the energy emission spectrum of the GQDs. The blue arrow is indicating changes in the maximum absorption peak, the corresponding increase of overlapping grade, in agreement with the extent of quenching from Figure S2.

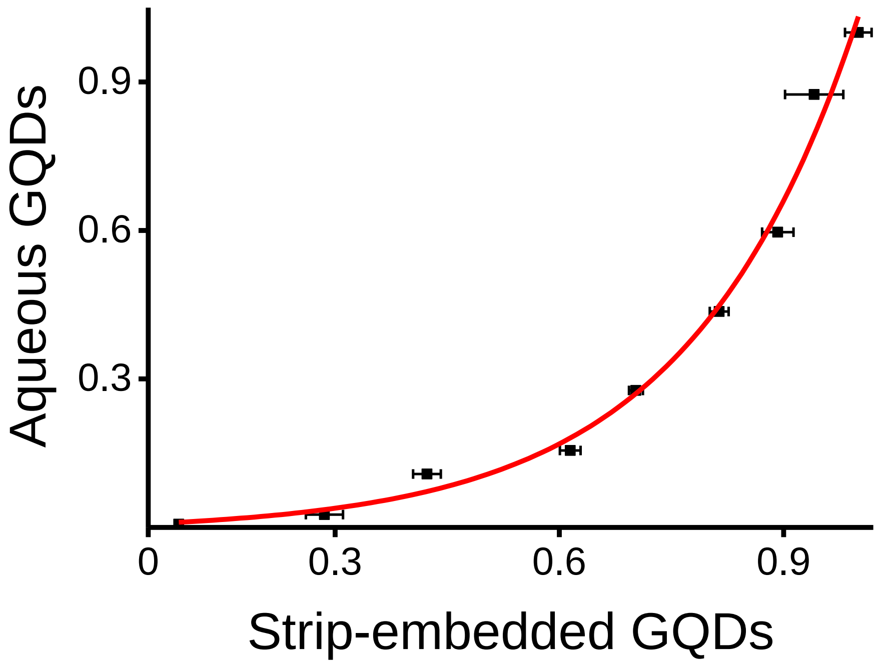

Figure S4. Correlation between the normalized intensity of aqueous GQDs and strip-embedded GODs.

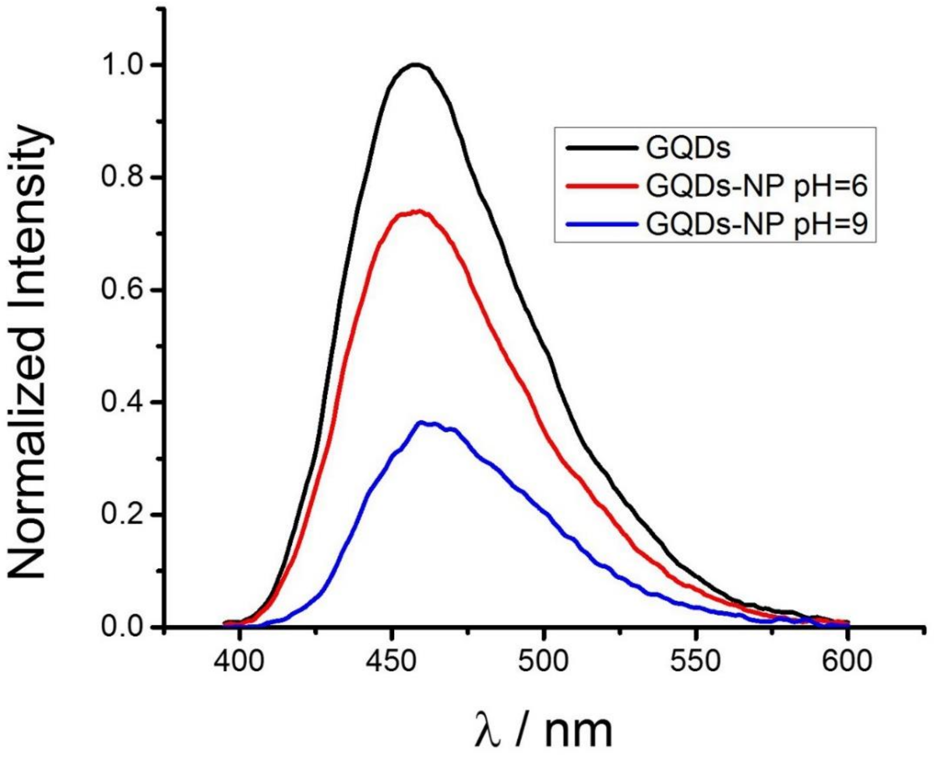

Figure S5. Quenching of the GQDs with 4-nitrophenol at different pH values.

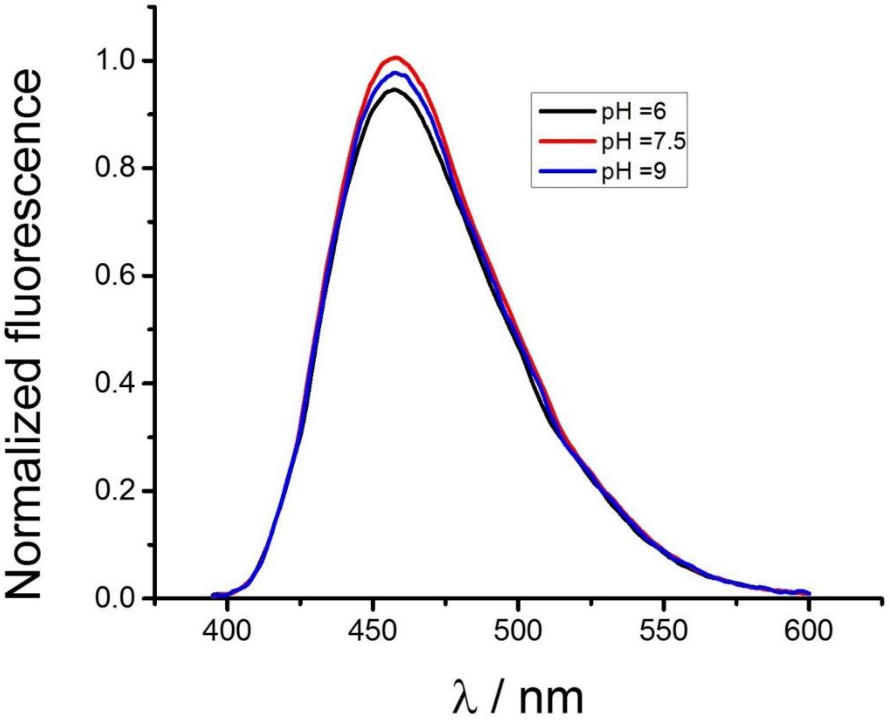

Figure S6. Emission spectrum of GQDs at different pH values
